# A pan-cancer study of selenoprotein genes as promising targets for cancer therapy

**DOI:** 10.1186/s12920-021-00930-1

**Published:** 2021-03-11

**Authors:** Wentao Wu, Daning Li, Xiaojie Feng, Fanfan Zhao, Chengzhuo Li, Shuai Zheng, Jun Lyu

**Affiliations:** 1 Clinical Research Center, The First Affiliated Hospital of Xi’an Jiaotong University, Xi’an, Shaanxi China; 2 Department of Clinical Research, The First Affiliated Hospital of Jinan University, 613 Whampoa Avenue, Tianhe District, Guangzhou China; 3 School of Public Health, Xi’an Jiaotong University Health Science Center, Xi’an, Shaanxi China

**Keywords:** Selenoprotein genes, GPXs, TXNRDs, Pan-cancer, Drug sensitivity, Therapeutic targets

## Abstract

**Background:**

The most important health benefit of selenium (Se) is in the prevention and control of cancer. Glutathione peroxidases (GPXs) and thioredoxin reductases (TXNRDs) are selenoenzymes that are thought to play a role in oxidative stress. The differential expression of genes of the TXNRD and GPX families is closely related to carcinogenesis and the occurrence of cancer. This study comprehensively analyzed the expression profiles of seven genes in the TXNRD and GPX families, in terms of their correlations with patient survival and immune-cell subtypes, tumor microenvironment, and drug sensitivity.

**Results:**

The expression profiles of genes in the TXNRD and GPX families differ between different types of cancer, and also between and within individual cancer cases. The expression levels of the seven analyzed genes are related to the overall survival of patients. The TXNRD1 and TXNRD3 genes are mainly related to poor prognoses, while other genes are related to good or poor prognoses depending on the type of cancer. All of the genes were found to be correlated to varying degrees with immune-cell subtypes, level of mechanistic cell infiltration, and tumor cell stemness. The TXNRD1, GPX1, and GPX2 genes may exert dual effects in tumor mutagenesis and development, while the TXNRD1, GPX1, GPX2, and GPX3 genes were found to be related to drug sensitivity or the formation of drug resistance.

**Conclusions:**

The results will greatly help in identifying the association between genes and tumorigenesis, especially in the immune response, tumor microenvironment, and drug resistance, and very important when attempting to identify new therapeutic targets.

**Supplementary Information:**

The online version contains supplementary material available at 10.1186/s12920-021-00930-1.

## Background

Selenium (Se) is found in almost all living things in nature, and is one of the trace elements that is essential for human survival. Although the bioavailability of Se varies among different types of organisms, it appears that its essential roles in biology—including its benefits to human health—are mainly due to its presence in proteins as selenocysteine [1–4]. The main role of selenocysteine is participating in the redox catalytic process. Se is thought to play a crucial role in regulating various pathophysiological processes in humans, including maintaining the cellular redox balance, development, immunity, reproductive health, and thyroid hormone metabolism [5]. Se is therefore often used to prevent cardiovascular disease, treat certain endemic and muscular diseases, delay the onset of AIDS in HIV-positive patients, and control cancer [5].

The most significant health benefit of Se is preventing and controlling cancer. Se exerts its biological effect through several selenoproteins. The main way that selenoproteins can exert anticancer effects is via its direct and indirect antioxidant properties. Glutathione peroxidases (GPXs) and thioredoxin reductases (TXNRDs) are generally considered to exert antitumor effects, since they block reactive oxygen species (ROS) produced by DNA-damaging H_2_O_2_ and lipid peroxides, and regulate the redox signaling system that plays a key role in the growth of many tumors [6, 7]. ROS are free radicals with unpaired electrons generated during normal physiological functions, and there is strong evidence that excessive ROS promotes carcinogenesis via increased oxidative stress and DNA mutation [8]. However, there is still no consensus conclusion on the circumstances under which different types of selenoproteins prevent or enhance carcinogenesis, since the various epidemiological, clinical, and laboratory studies have produced conflicting results [5, 9–11]. Many selenoproteins have been found to be associated with the occurrence and poor prognosis of cancer, which means that selenoprotein may exert contrasting promotion and suppression effects on tumors under different circumstances [7]. In addition, it is worth noting that Se status determines selenoprotein expression, thus affecting the risk of developing cancer when Se status is sub-optimal [12, 13]; whereas in cancer patients, selenoprotein expression is not only affected by Se status but also by the tumour microenvironment [14]. A correct understanding of the correlation between tumor microenvironment and selenoprotein expression level will help to further explore the potential value of selenoprotein in tumor therapy.

Understanding the relationship between TXNRDs and GPXs and the occurrence and prognosis of cancer would be helpful when attempting to treat malignant tumors and discover new therapeutic targets. Although previous studies have suggested that Se-related proteins such as GPXs and TXNRDs may be associated with tumor development, possibly in a bidirectional manner, previous studies have only investigated the effects of certain genes in certain types of cancer, mostly using cell lines and animal models [4, 15]—there has been no systematic study on these two Se-related genes in humans.

This study used pan-cancer data from The Cancer Genome Atlas (TCGA) to investigate the expression patterns of members of the TXNRD and GPX gene families (TXNRD1, TXNRD2, TXNRD3, GPX1, GPX2, GPX3, and GPX4 genes) and their association with primary overall survival in 33 types of cancer, and to correlate their expression levels with the tumor microenvironment and pharmacological activity.

## Results

### Pan-cancer expression patterns of TXNRD and GPX genes

**Fig. 1:**
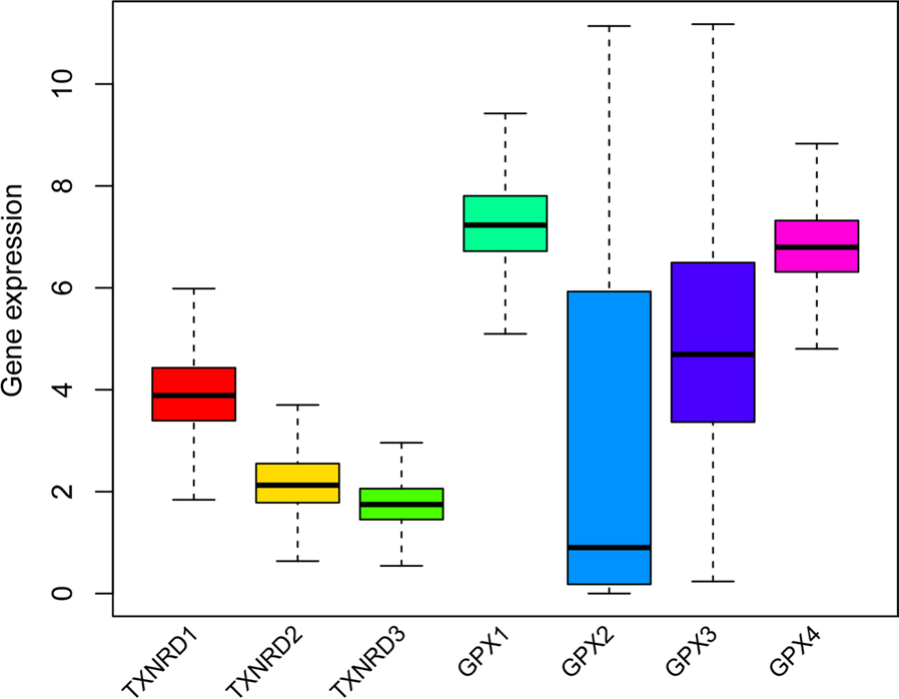
Distribution of TXNRD and GPX genes expression across all 33 cancer types

**Fig. 2:**
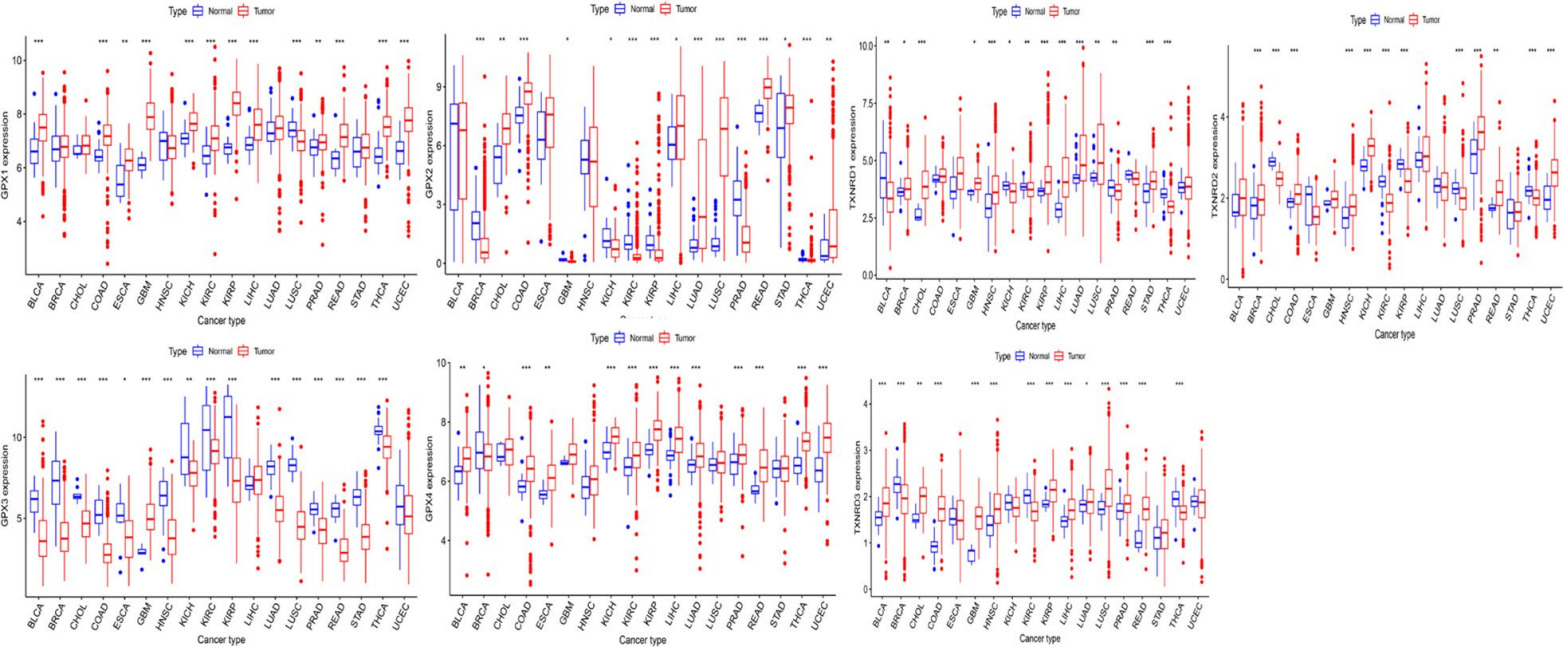
Expression levels of TXNRD and GPX genes in cancerous and adjacent normal tissues for 18 cancer types that have more than 5 adjacent normal samples

We examined the expression patterns of TXNRD and GPX genes in TCGA pan-cancer data. The gene expression level in the TXNRD family was highest for TXNRD1 and lowest for TXNRD3, while in the GPX family it was highest for GPX1 and GPX4, and lowest for GPX2 (Fig. 1). Figure 2 shows the expression level for each specific gene in different types of cancer as well as the difference in expression between normal and tumor samples in different types of cancer. The expression levels of genes in the TXNRD and GPX families differed between normal samples and most types of cancer samples, but this was not the case for ESCA, in which the three genes of the TXNRD family. There were also differences in the expression of the same gene in different types of cancer. Some genes were expressed at similar levels in different types of cancer, including GPX1, GPX4, TXNRD1, TXNRD2 and TXNRD3. It can be seen that these genes were ubiquitously expressed in different types of cancer. In contrast, other genes were specifically expressed. For example, GPX2 is mainly expressed in the gastrointestinal tract, and the three cancers with the highest expression levels were COAD, READ, and STAD, while the other cancers showed a trend of low expression especially in the GBM, KIRC, KIRP, and THCA. Similarly, GPX3 was highly expressed in KICH, KIRP, KIRC and THCA, indicating that GPX3 is mainly expressed in the kidney and thyroid. The above findings suggested that gene expression in the TXNRD and GPX families differs between different tumors, and there was also heterogeneity among members within each family. Therefore, when studying the relationship between genes and tumors, it is necessary to study each gene independently and consider the specific expression of some genes. Previous studies have found that gene expression disorders are common in tumors, which was also reflected in our study. The GPX3 gene tended to be down-regulated in all types of cancers except for GBM, while the other six genes were up-regulated or down-regulated in the different types of cancer.

**Fig. 3:**
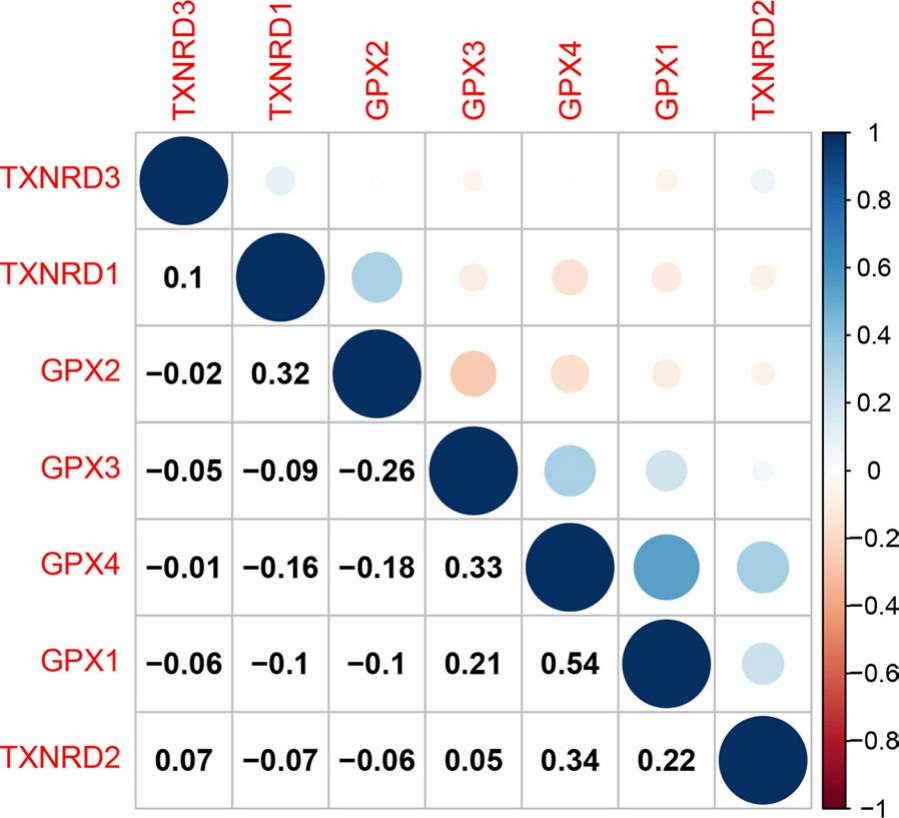
Correlation plot based on Spearman Correlation test results to show the correlation of gene expression among the TXNRD and GPX family members across all 33 cancer types

Figure 3 shows the correlations between the expression levels of the seven genes analyzed in this study. The presence of a positive correlation between two genes may indicate commonality in structure or function, while a negative correlation indicates that the functions of the two genes have potential antagonistic effects. We found that the positive correlation was strongest between the GPX4 and GPX1 genes (r = 0.54, *p* < 0.001), while the negative correlation was strongest between the GPX2 and GPX3 genes (r = –0.26, *p* < 0.001).

### Expression patterns of TXNRD and GPX genes were related to overall survival

**Fig. 4:**
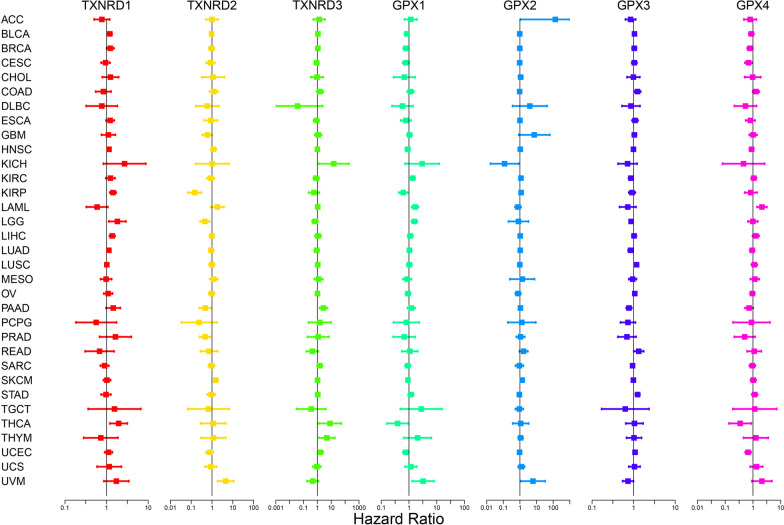
Association of TXNRD and GPX gene expression with patient overall survival for different cancer types

To investigate the relationships between gene expression levels and overall survival, we performed survival analyses of the seven genes from the two gene families to predict whether the expression of specific genes promoted or inhibited cancer. The results obtained from Cox proportional-hazards models are presented in forest plots in Fig. 4. The expression levels of all genes in the TXNRD and GPX families were related to the overall survival of patients, whereas their relationships with good and poor prognoses varied with the specific gene and type of cancer. The expression levels of the TXNRD1 and TXNRD3 genes were mainly related to a poor cancer prognosis. TXNRD1 gene expression predicted low survival rates for BLCA, BRCA, HNSC, LGG, LUAD, and THCA, while TXNRD3 gene expression predicted low survival rates for KICH, PAAD, THCA, THYM, and UCEC. The expression of the TXNRD2 gene was associated with poor prognoses of SKCM and UVM, in contrast to better survival in LGG, KIRP, PAAD and PRAD. Each member of the GPX family was associated with both good and poor prognoses. Specifically, GPX1 gene expression was associated with good prognoses of BRCA, KIRP, THCA, and UCEC, but poor prognoses of KIRC, LAML, LGG, and UVM. The GPX2 gene was associated with a good prognosis in KICH but poor prognoses of ACC, KIRP, and UVM. The GPX3 gene improved the survival of KIRC, LGG, LUAD, PAAD, and UVM, but was predictive of poor prognoses of COAD, LUSC, READ, and STAD. Finally, the GPX4 gene was associated with a poor prognosis of LAML, but had survival benefits in BRCA, CESC, THCA, and UCEC. The numerical values of overall hazard ratio for the different genes were showed in Additional file 1: Table S1.

### TXNRD and GPX genes are associated with immune response and the tumor microenvironment in cancer

**Fig. 5:**
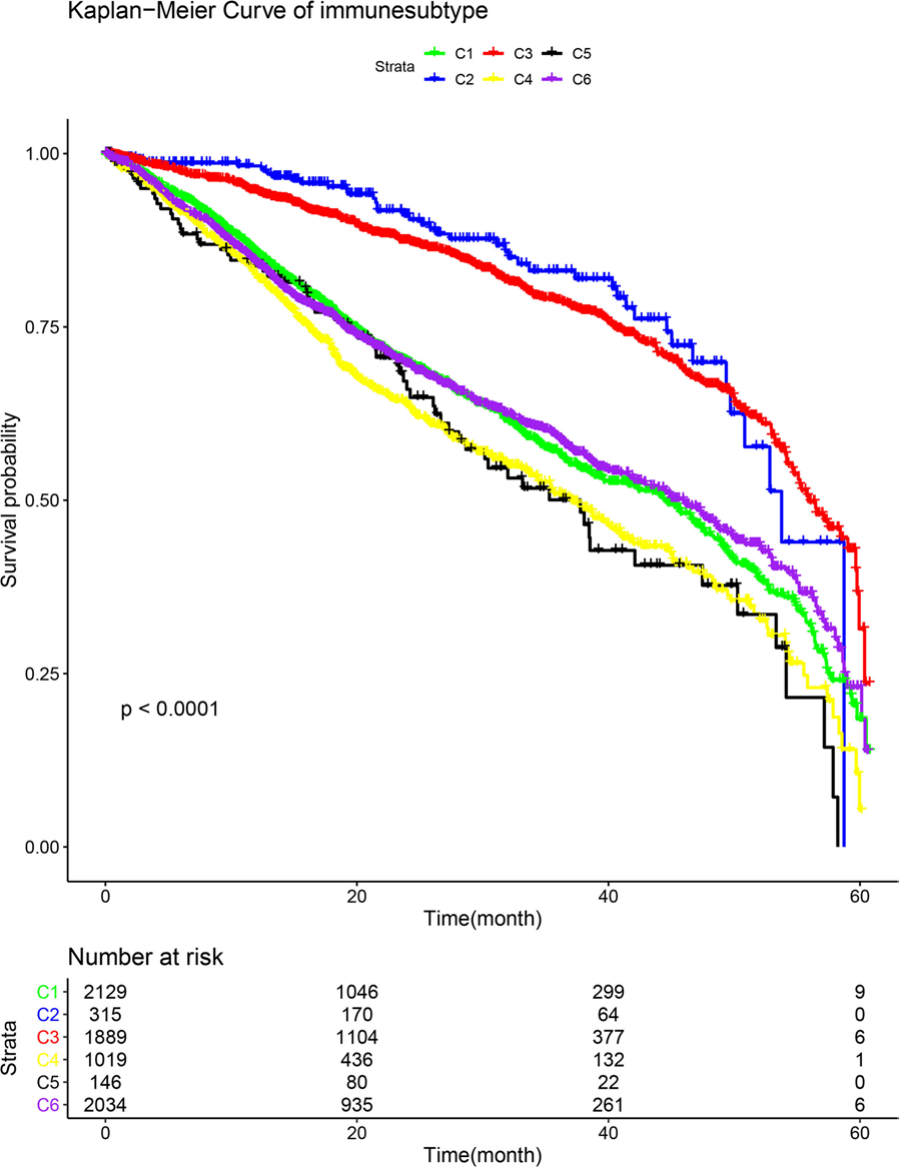
Survival plot of patients belonging to different immune subtypes

**Fig. 6:**
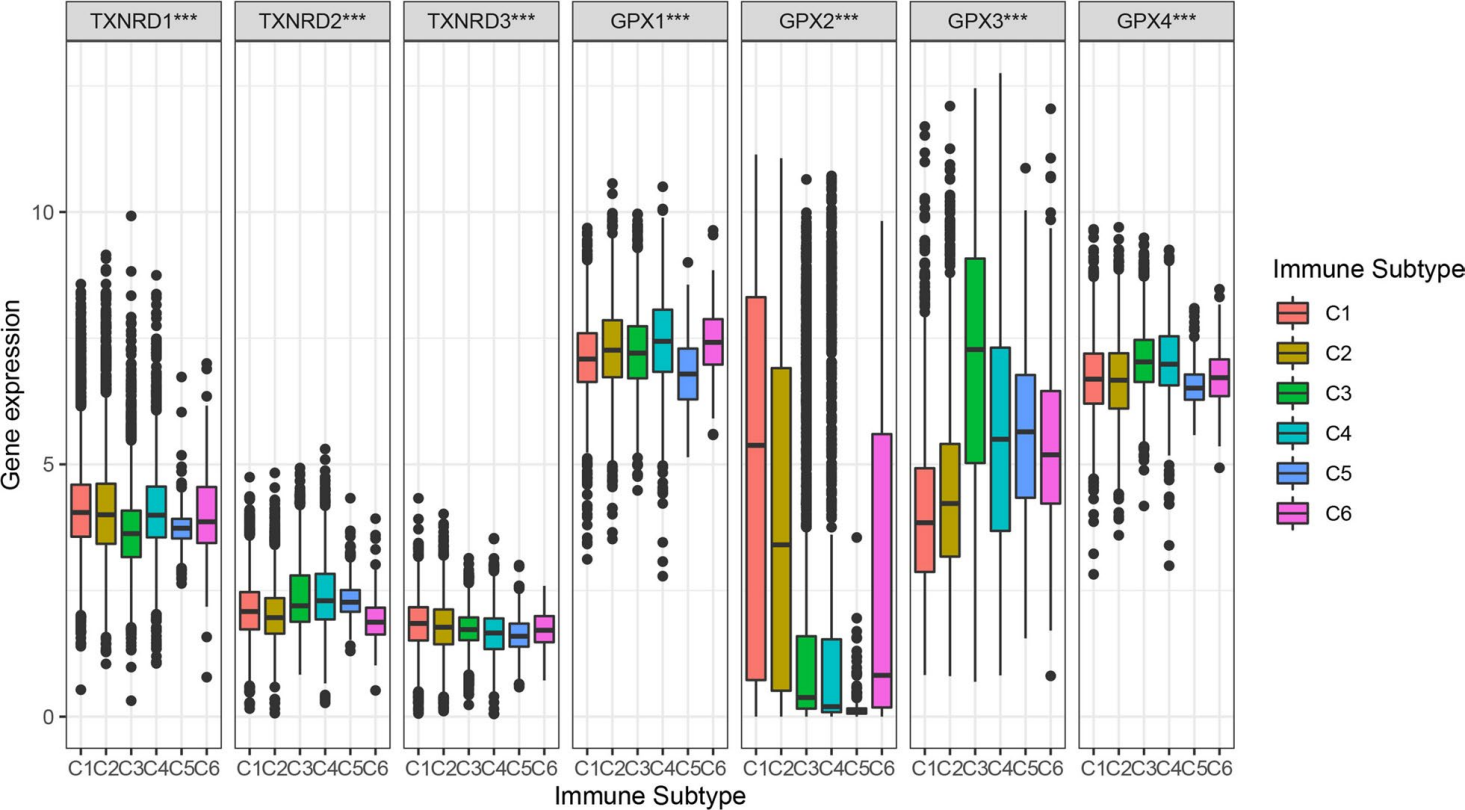
Association of TXNRD and GPX genes expression with immune infiltrate subtypes across all the cancer types

Our analysis of the relationships between different immune-cell subtypes (C1 to C6) and the overall survival of all patients revealed that patients with immune-cell subtypes C2 and C3 had higher survival rates, while those with immune-cell subtypes C1, C4, C5, and C6 had poor prognoses (Fig. 5). Further, we analyzed the correlations between gene expression and different immune-cell subtypes (Fig. 6). The TXNRD1, TXNRD2, and TXNRD3 genes were strongly expressed in the C1 and C4, C4 and C5, and C1 and C6 subtypes, respectively. These genes were related to poor prognoses, which suggests that they play roles as tumor promotors. Similarly, the significant correlation between high expression levels of the GPX1 gene and the C4 and C6 subtypes suggest that these genes play a role as tumor promotors, since patients with these immune-cell subtypes have lower survival rates. In contrast, the expression level of the GPX3 gene was far higher in the C3 subtype than in the other subtypes, while a higher expression level of the GPX4 gene was also correlated with the C3 subtype, suggesting that the strong expression of these genes is related to strong immunity; that is, these genes may play a major role in inhibiting cancer. The expression level of the GPX2 gene was much higher in the C1 and C2 subtypes than in the other subtypes.

**Fig. 7:**
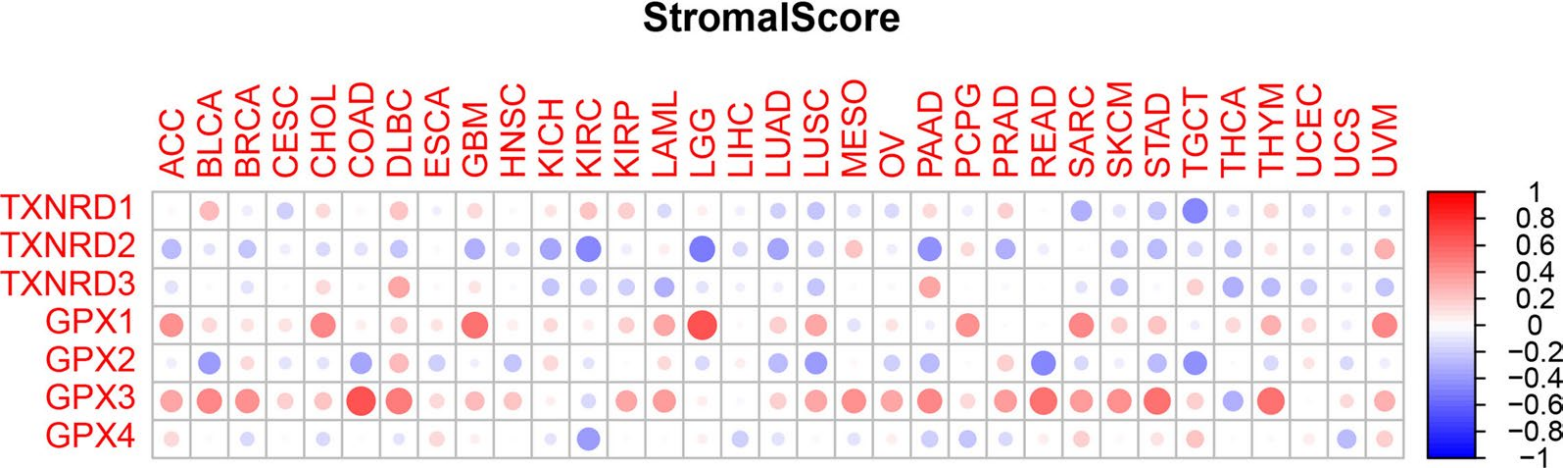
Association between TXNRD and GPX genes expression and stromal scores of 33 different cancer types based on ESTIMATE algorithm

**Fig. 8:**
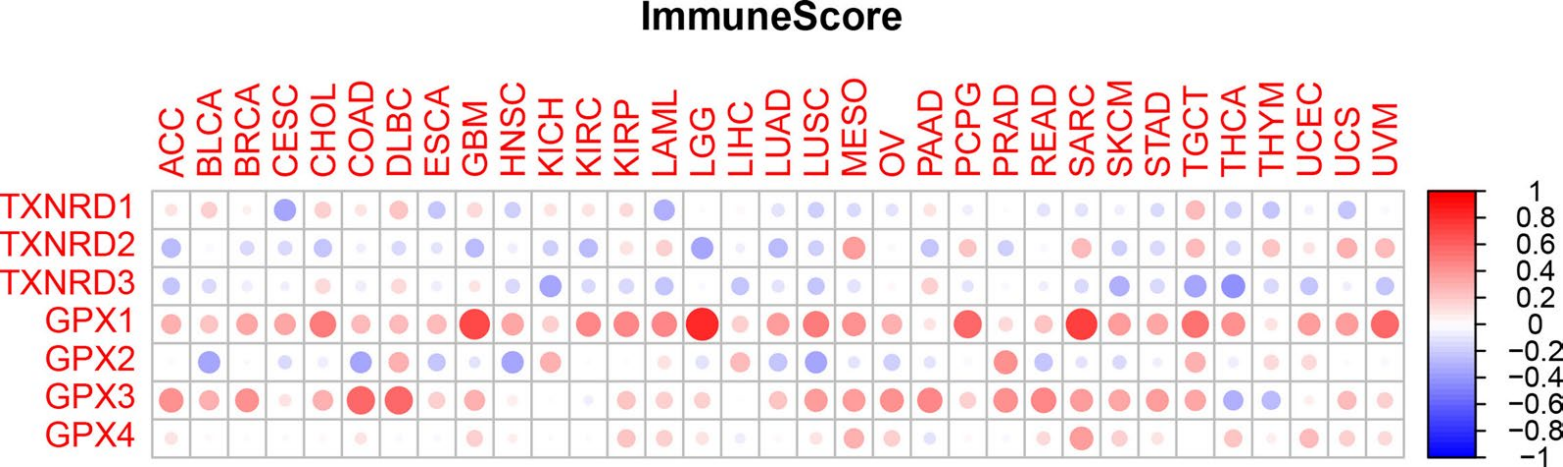
Association between TXNRD and GPX genes expression and immune scores of 33 different cancer types based on ESTIMATE algorithm

Macrophages and fibroblasts also play important roles in the development of tumors [16], and so we investigated the relationships between gene expression levels and the stromal- and immune-cell scores as calculated using the ESTIMATE algorithm. Although there were strong correlations between each gene and the stromal- and immune-cell scores, there was considerable heterogeneity between the different genes and also the same gene among different cancers. The expression levels of the GPX1 and GPX3 genes were positively correlated with the stromal- and immune-cell scores, and the TXNRD2 gene was negatively correlated with the stromal-cell score for all but a few cancers. Other genes had both positive and negative associations with stromal-cell scores, depending on the type of cancer; the details are provided in Figs. 7 and 8. It is worth mentioning that the GPX3 gene had the strongest correlation with the stromal-cell score (r = 0.65, *p* < 0.001), while the GPX1 gene had the strongest correlation with the immune-cell score (r = 0.69, *p* < 0.001).

### Relationships between gene expression and tumor stemness and chemotherapeutic drug sensitivity

CSCs are a subset of the cancer cell population that possess self-renewal ability and account for the initiation, progression, metastasis, and recurrence of tumors [17]. The three most widely recognized characteristics of CSCs are their carcinogenicity, association with tumor metastasis, and involvement in the development of drug resistance [18, 19]. Tumor stemness was measured in the present study using the RNA stemness score based on RNAss and the DNA stemness score based on DNAss.

**Fig. 9:**
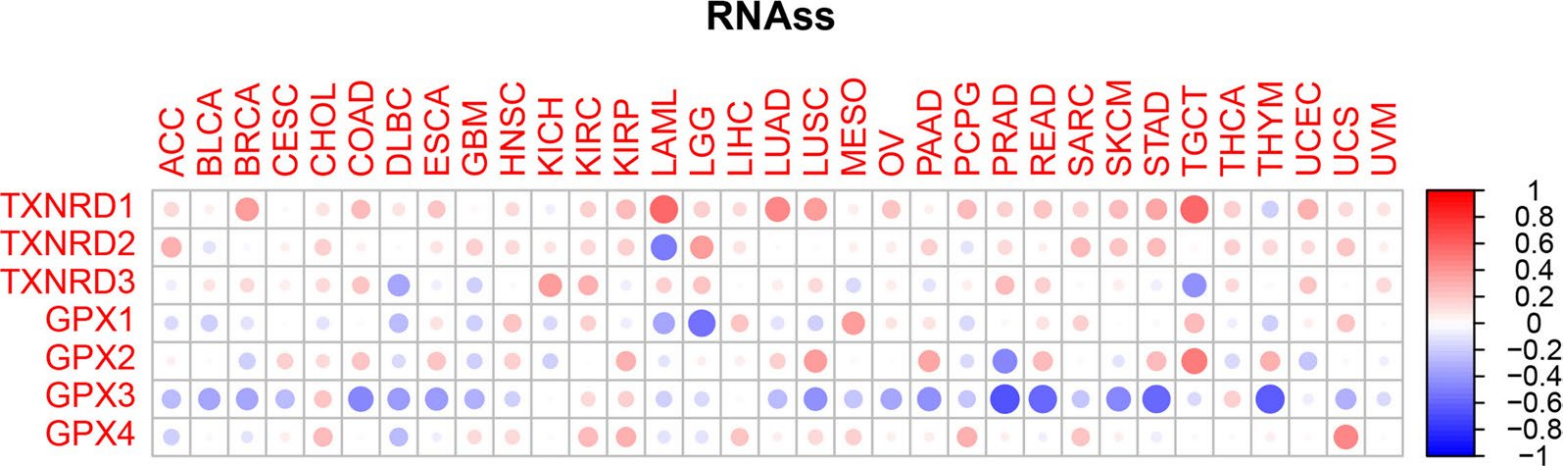
Association of TXNRD and GPX genes expression with RNAss

**Fig. 10:**
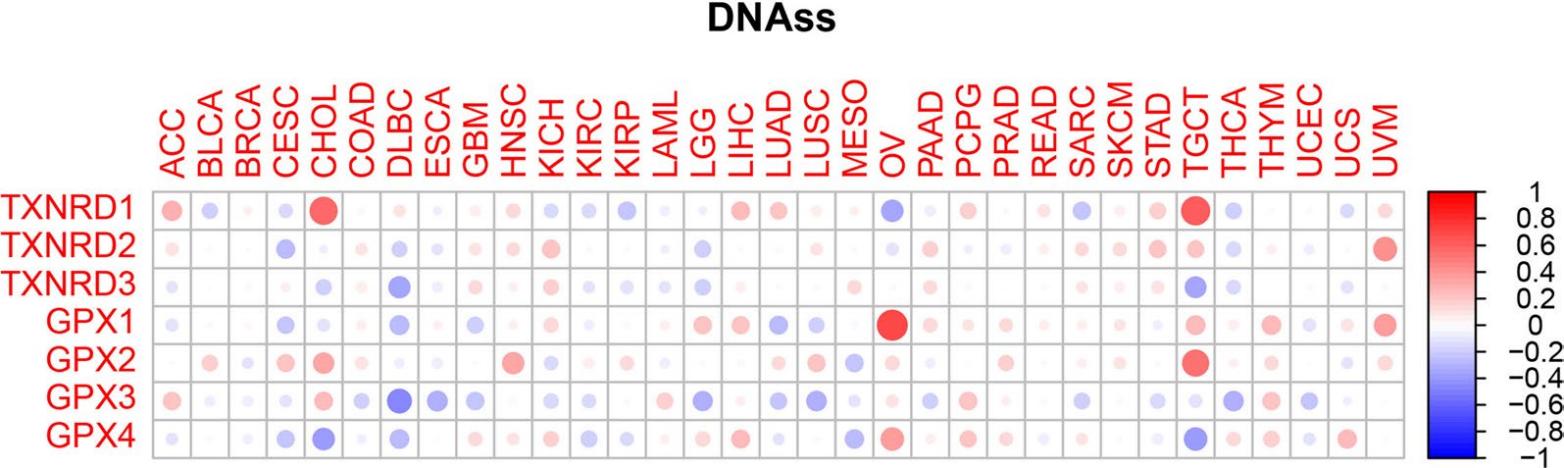
Association of TXNRD and GPX genes expression with DNAss

Figures 9 and 10 present the correlations between members of the TXNRD gene family and members of the GPX family with RNAss and DNAss in different types of cancer. The GPX3 gene had the strongest negative correlation with RNAss (r = –0.65, *p* < 0.001), while the TXNRD1 gene had the strongest positive correlation with RNAss (r = 0.63, *p* < 0.001); these two genes also had the strongest negative and positive correlations with DNASS (r = –0.44 and *p* = 0.002, and r = 0.63 and *p* < 0.001, respectively; only genes with statistically significant associations with RNAss or DNAss were compared). In all types of cancer, the GPX3 gene was mainly negatively correlated with RNAss, which this conclusion also made for the correlation between this gene and DNAss. It was particularly interesting that the three genes of the TXNRD family showed significant positive correlations with RNAss for LGG, PRAD, THCA, and UECE, while the four genes of the GPX family showed a significant negative correlation with RNAss for BRCA. However, we did not find either similar or statistically significant results in investigations of the associations of these genes with DNAss. These contradictory results suggest that RNAss and DNAss can be used to identify distinct cancerous cell populations characterized by different features or degrees of stemness in different cancers [20].

**Fig. 11:**
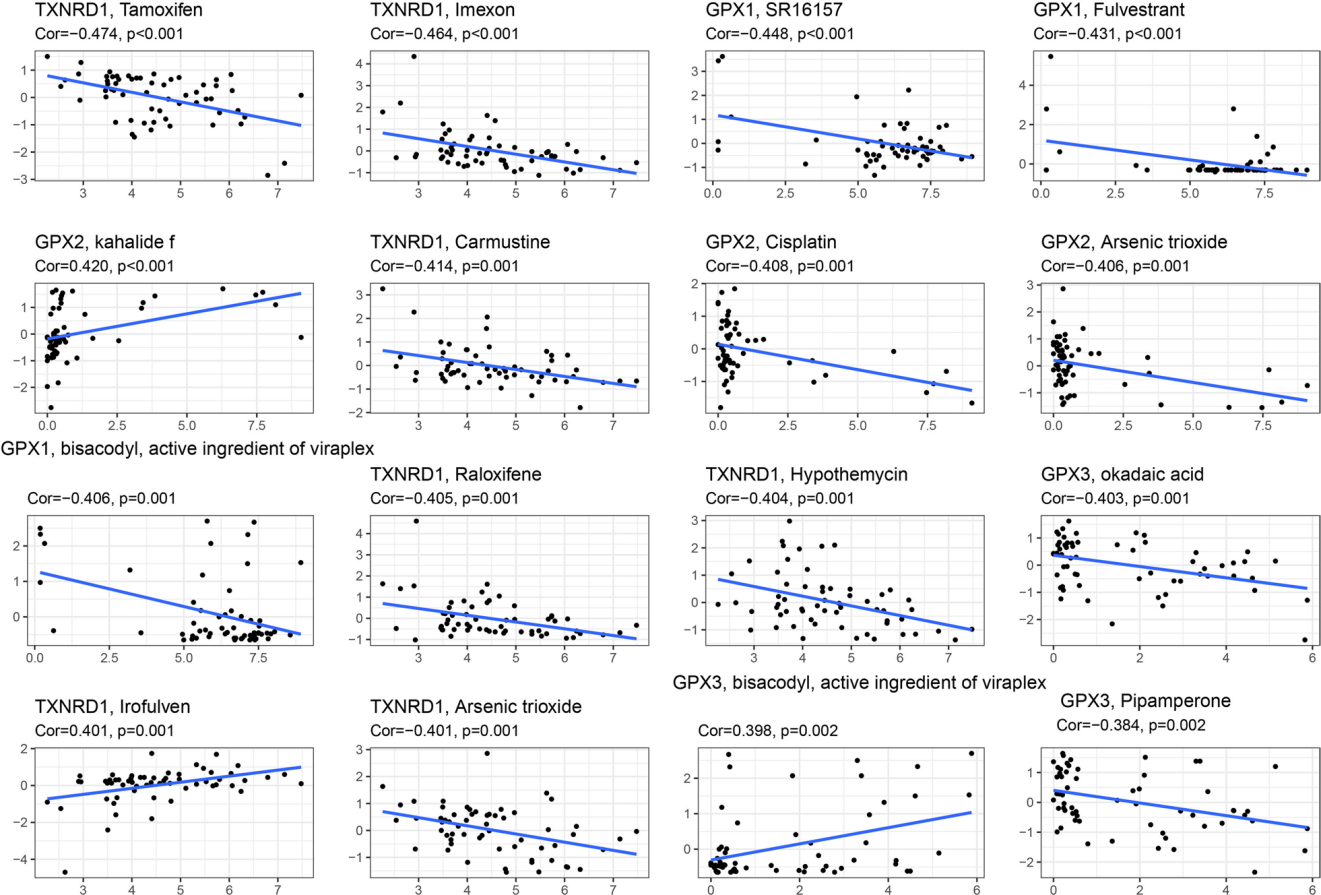
Association of TXNRD and GPX genes expression with drug sensitivity

We further investigated the relationships between the expression levels of genes in the TXNRD and GPX families and more than 200 existing chemotherapy drugs in 60 human cancer cell lines. Figure 11 shows the statistically significant results. The expression of the GPX1 gene was associated with increased drug resistance in various cell lines, including SR16157 (for treating breast cancer), fulvestrant (for treating for breast cancer), and bisacodyl active ingredient (for treating glioblastoma). The expression of the TXNRD1 gene was also associated with increases in the resistance of multiple drugs, including tamoxifen (for treating breast cancer), imexon (for treating skin cancer), carmustine (for treating osteoma and non-small-cell lung cancer), raloxifene (for treating breast cancer), hypothemycin (for treating thyroid cancer, colon cancer, and melanoma), and arsenic trioxide (for treating leukemia). On the other hand, TXNRD1 gene expression also increased the sensitivity of cell lines to irofulven, which is used to treat ovarian and prostate cancer.

The GPX2 and GPX3 genes also exerted different effects for different drugs. The GPX2 gene increased the sensitivity of cell lines to kahalide (for treating breast cancer) and also increased the tolerance of cell lines to cisplatin (for treating breast cancer, bladder cancer, esophageal cancer, and head and neck cancer) and arsenic trioxide (for treating leukemia). The GPX3 gene increased the sensitivity of cell lines to bisacodyl active ingredient (for treating glioblastoma) and also increased the tolerance to okadaic acid (for treating colon cancer).

The observed genetic heterogeneity for the effects of different drugs indicates that the presence of different genes may result in the same drugs producing either the same or entirely different functions, such as the TXNRD1 and GPX2 genes enhancing cell lines with arsenic trioxide resistance, the GPX1 gene increasing the resistance of cell lines to the bisacodyl active ingredient, and the GPX3 gene increasing the sensitivity of the cell lines to bisacodyl. These findings suggest that if TXNRD and GPX family genes are used as tumor therapy targets, the synergistic or antagonistic effects between these genes and existing chemotherapy drugs should also be taken into account, so as to maximize the benefits to patients.

## Discussion

The relationship between Se and cancer was one of the first findings in the history of multifaceted studies of Se, and has major health implications. Many epidemiological studies, cell-line studies, and animal models have found a strong association between Se and cancer [4]. In the context of the human spectrum of diseases shifting toward chronic diseases and cancer, as well as the deepening of research into cancer in humans, Se is more likely to have potential in cancer treatments [21]. Two Se-containing enzymes (TXNRD and GPX) have attracted considerable attention due to their role in oxidative stress. Based on this, the present study has conducted the first pan-cancer analysis of TXNRD and GPX genes.

We observed that the expression levels of TXNRD and GPX genes and the heterogeneity of their expression patterns differed in normal samples and in tumor samples of the same type. In general, the GPX3 gene showed a down-regulation trend in all types of cancers except for GBM, while the other six genes were up-regulated or down-regulated according to the specific type of cancer. The ubiquitously expression of GPX1,GPX4,TXNRD1 and TXNRD2 in humans has been reported in previous studies, and our study further confirmed these genes were also ubiquitously expressed in different types of cancer [22–24]. GPX2 is present in the cytoplasm and nucleus of cells from the gastrointestinal tract and therefore mainly expressed in this organ [25, 26]. We found that GPX3 was highly expressed in KICH, KIRP, KIRC and THCA. In fact, GPX3 is an extracellular protein that is synthesized in the kidney and also produced by thyroid follicular cells [27]. Previous studies reported that TXNRD3 is expressed specifically in the testis [28, 29]. In our study, we were limited by the number of samples of testicular germ cell tumors in the TCGA database, so we were unable to verify the expression level of TXNRD3 in testicular germ cell tumors. But we did find that TXNRD3 lowly expressed in other types of cancer. Survival analysis revealed that the expression levels of these genes were closely related to patient survival, but the directions of the associations differed between different types of cancer, except for the TXNRD1 and TXNRD3 genes, which showed significant adverse prognoses in all cancer types.

Immune-cell subtypes in the tumor microenvironment are closely related to the prognosis of patients. We found that the expression levels of TXNRD and GPX genes differed between immune-cell subtypes. For example, three genes of the TXNRD family were associated with more aggressive immune-cell subtypes, suggesting a poor prognosis, while GPX3 gene expression was stronger in the C3 subtype than in the other subtypes, indicating a possible correlation with a good prognosis. Previous studies have found that TXNRD and GPX genes play regulatory roles in inflammation and immunity [30], and these correlations were confirmed in the present study. These results provide clues for identifying new cancer therapeutic targets and for predicting the efficacy of immune checkpoint modulators in cancer patients. The GPX3 and TXNRD1 genes had the strongest negative and positive correlation with the tumor stemness, respectively. We can reasonably infer that the GPX3 gene mainly plays an inhibitory role in the process of tumor occurrence and development, while the TXNRD1 gene mainly plays a promoting role. Finally, by testing the relationship between the expression levels of these two families of selenoprotein genes in NCI-60 cell lines and drug sensitivity, we found that the TXNRD1, GPX1, GPX2, and GPX3 genes may play a role in the drug sensitivity or drug resistance of cancer cells.

The effects on cancer and the potential in new anticancer treatments have been the discussed most widely for the TXNRD1 gene from among the TXNRD family members. This is because the selenocysteine residues at the c-terminal active site of the TXNRD1 gene are easily accessible and have special reactivity, which make targeted regulation easier [31]. The present study found that the TXNRD1 gene was often strongly expressed in cancer samples, and both the survival analyses and tumor stemness results suggested that the expression level of the TXNRD1 gene is related to poor prognoses in patients and that this gene plays a promoter role in the process of tumor occurrence. These results are consistent with many previous reports [32]. Combined with it being relatively easy to target and control the TXNRD1 gene, we believe this gene to be a promising cancer therapeutic target, and that its targeted inhibition may be effective in treating cancer.

Some possible inferences about the beneficial effects and internal mechanisms of the TXNRD1-gene-targeting drugs in anticancer treatment include the activation effect of a TXNRD1 inhibitor on nuclear factor (erythroid-derived 2)-like 2 (Nrf2) demonstrated in previous studies [33]. The idea that cancer cells face increases in their own oxidative stress due to dysfunctional energy metabolism, proliferation drive, and abnormal cell phenotypes is rapidly becoming widely accepted [34–37]. A TXNRD1 inhibitor can increase the ROS level of cancer cells and induce cancer cell death by inhibiting TXNRD1 activity [38, 39]. Some studies have shown that normal cells can survive after TXNRD1 activity is inhibited, suggesting that a TXNRD1 inhibitor can kill cancer cells directly and selectively [40–42]. In addition, many TXNRD1-inhibiting drugs can simultaneously activate Nrf2 [33], which helps to protect normal cells from oxidative damage so as to inhibit their induction and transformation associated with cancer [43, 44]. The redox system obviously affects many different cellular signaling pathways, and we note that the inactivation of the TXNRD1 gene may lead to impaired function of P53, thereby increasing the probability of cancer [45]. Although some studies have found that normal cells are more resistant to TXNRD1-targeted inhibition, there is still insufficient evidence from in vivo and clinical trials, and high levels of ROS may contribute to cancer mutagenesis. Therefore, further investigations are needed into the role of TXNRD1-targeted inhibitors on cancer and the underlying mechanisms.

Meanwhile, many clinical therapies including radiotherapy and chemotherapy induce oxidative stress in cells [46, 47], and so the effects of oxidative stress should also be considered when applying the currently available treatment modalities. In addition, we observed that the TXNRD1 gene was associated with increased tolerance to various chemotherapy drugs. Only a few studies have investigated the relationships of the expression levels of TXNRD2 and TXNRD3 genes with tumors, with most finding positive correlations [48, 49]. Our study similarly found up-regulation in the TXNRD2 and TXNRD3 genes in various types of cancer, which was also correlated with a poor prognosis of immune-cell subtypes. The positive correlation between the TXNRD2 gene and RNAss means that this gene may play a role in tumor promotor. The survival analysis showed that the TXNRD2 gene was associated with poor prognoses of SKCM and UVM, but good prognoses of LGG, KIRP, PAAD, and PRAD, while the TXNRD3 gene was correlated with poor prognoses of KICH, PAAD, THCA, THYM, and UCEC. We believe that the relationships between the TXNRD2 and TXNRD3 genes and tumors are associated with the roles that these genes play in cellular oxidative stress. Whether TXNRD2 and TXNRD3 can serve as promising cancer treatment targets requires further analysis of how these genes play a role in oxidative stress in different cancers.

In contrast to the other genes in the GPX family, the GPX3 gene had a low expression level in all types of tumors, while it was also observed to be associated with better immune-cell subtypes and negatively with tumor stemness; this gene might therefore be a novel cancer inhibitor. Many studies have found that the down-regulation of the GPX3 gene in various cancers is caused by promoter methylation. The anticancer activity of the gene is associated with ROS inactivation, which protects cells from genetic mutations and cancer-related protein oxidation [50, 51]. Recent studies have elaborated on the tumor suppressor mechanism of the GPX3 gene. Yan et al. demonstrated that this gene suppresses prostate cancer by inhibiting c-Met expression [50], Qi et al. reported that the GPX3 gene inhibits the invasion of hepatocellular carcinoma cells [52], while An et al. investigated the mechanism of the GPX3-gene-mediated inhibition of proliferation of lung cancer cells, and showed that this gene inactivates ROS, thereby inhibiting the ERK–NF-κB–cyclin B1 signaling pathways and leading to cell-cycle arrest to cancer inhibition [53]. The GPX3 gene is therefore likely to be a promising target for cancer therapy, but more robust evidence is needed to elucidate its anticancer mechanisms and the conditions in which it can be applied. In addition, the possibility that the GPX3 gene may increase chemotherapeutic drug resistance also needs to be reconsidered, since other studies—like the present one—have found that this gene may be associated with increased drug resistance (Fig. 11) [54, 55].

The GPX1 gene is able to prevent oxidative DNA mutations [56] and counteracts the production of COX/LOX-derived proinflammatory mediators such as prostaglandins and leukotrienes [5]. This could explain the significant positive correlation between the GPX1 gene and stromal- and immune-cell scores in multiple tumor types. In other words, this gene is involved in both stromal- and immune-cell activities, and therefore may prevent carcinogenesis at least during the initiation phase. Our study found that GPX1 gene expression tended to be elevated in most cancers, and the results of survival and tumor stemness analyses suggested that the GPX1 gene plays different roles in different types of cancer. This conclusion is consistent with previous findings, and so the role of the GPX1 gene in cancer might need to be analyzed while considering specific types of cancer in order to identify the underlying mechanism [57]. However, the GPX1 gene is definitely becoming more widely accepted as a potential biomarker for diagnosis and prognosis [57].

Previous studies have suggested that high GPX2 gene expression is associated with a poor cancer prognosis, which appears to be supported by the present finding of a significant negative correlation between the GPX2 and GPX3 genes. The survival analysis found that the GPX2 gene was associated with poor prognoses of ACC, KIRP, and UVM, but with a good prognosis of KICH. The GPX2 gene was strongly expressed in both the C2 subtype associated with a good prognosis and the C1 subtype associated with a poor prognosis.

Such a dual role of the GPX2 gene in cancer has recently been widely discussed, with it specifically being shown to reduce the levels of H_2_O_2_ and free radicals in normal cells and being reported as an anticancer enzyme [55]. The mutation of normal cells into cancer cells might reverse the basic physiological functions associated with the GPX2 gene [58]. Our study also found a positive correlation between the GPX2 gene and RNAss, suggesting that the expression of this gene in tumor cells is associated with a poor prognosis. The survival analysis further showed that GPX2 gene expression is a risk factor for survival in ACC, KIRP, and UVM. Previous studies have also shown that this gene is associated with the progression of malignant tumors [59], and animal studies of breast and liver cancer have elucidated the molecular mechanisms via which the gene regulates tumor proliferation [60, 61], suggesting that it is a significant therapeutic target for tumors. In addition to the mechanisms underlying the dual role of the GPX2 gene in tumorigenesis and proliferation remaining unclear for some cancers, the association of this gene with increased drug resistance may also be a challenge for its use as a new cancer treatment target, particularly regarding its observed association with increased cisplatin resistance.

It has been reported that the GPX4 gene inhibits ferroptosis in cancer cells [62], as also found in the present survival analysis. At the same time, we found a strong positive correlation between the GPX4 and GPX1 genes, and we hypothesized that the latter plays an important role in regulating cell death and oxidation via its interaction with the former gene. One noteworthy issue is that the expression levels of both genes are associated with a poor prognosis in LAML, with similar conclusions made for previous studies involving the GPX4 gene and LAML [57]. Therefore, the role of the GPX1 and GPX4 genes in LAML may need to be reassessed and validated using additional clinical samples and animal trials.

This study utilized several online databases and the most popular bioinformatics theories to conduct a comprehensive analysis of the relationships between different genes and different tumors. The study was designed as a large-sample, low-cost, large-scale genomics research and functional analysis, but it was also subject to some limitations. First of all, all the samples involved in this study were from open online databases, so we were unable to control the experimental conditions. For example, we were unable to obtain the relevant information of Se level in the culture medium. Meanwhile, the absence of some data limited the scope of our analysis, such as the expression level of TXNRD3 in TGCT. Secondly, the conclusion of our study has not been verified by other external data sets, which also suggests that our next step should be to reasonably verify this conclusion with our own data sets or other public data sets. Finally, our conclusions are mainly drawn through pan-cancer analysis and bioinformatics analysis, which can only illustrate the statistical correlation but not the causal relationship. The present results from a pan-cancer analysis based on an online database need to be verified in laboratory analyses.

## Conclusion

Seven genes of the selenoprotein GPX and TXNRD families have been systematically summarized, and their association with different types of cancer, immune-cell subtypes, and molecular subtypes have been investigated using a pan-cancer methodology. Although our findings need further validation from laboratory results, they will greatly aid in identifying the roles of Se-related genes in tumorigenesis, especially in the immune response, tumor microenvironment, and drug resistance. This information with reveal possible therapeutic targets for malignant tumors, aid the development of personalized cancer therapies and provides new ideas for further research.

## Methods

### Collection of TCGA pan-cancer data and patient selection

TCGA is a project that began in 2005 and uses genome sequencing and bioinformatics to classify mutations associated with cancer. This project is supervised by the Cancer Genomics Center at the National Cancer Institute and the US-government-funded National Human Genome Institute (www.cancer.gov/about-nci/organization/ccg/research/structural-genomics/tcga). TCGA database currently contains information on more than 200 types of cancer and clinical patient information, which therefore represents a large data set for use in tumor genome analysis [63].

We downloaded TCGA pan-cancer data using open-source software, including clinical data, stemness scores based on mRNA expression (RNAss) and DNA methylation (DNAss), RNA-Seq (RNA SeqV2 RSEM), and immune-cell subtypes. The 11,057 obtained samples covered 33 different types of cancer, and comprised 10,327 tumor samples and 730 normal samples. The smallest number of tumor samples was 36 for CHOL, while the largest number was 1104 for BRCA. All of the tumor samples were obtained during the surgical resection of primary tumors that had received no prior neoadjuvant treatment. Detailed information about these samples is presented in Table 1.

**Table 1:** Summary of TCGA pan-cancer data

Primary disease type	TCGAID	Total N	Primary tumor	Normal tissue	OS_censored	OS_event
Adrenocortical cancer	ACC	79	79	0	61	34
Bladder urothelial carcinoma	BLCA	430	411	19	242	199
Breast invasive carcinoma	BRCA	1217	1104	113	1035	209
Cervical and endocervical cancer	CESC	309	306	3	227	77
Cholangiocarcinoma	CHOL	45	36	9	38	30
Colon adenocarcinoma	COAD	512	471	41	423	116
Diffuse large B-cell lymphoma	DLBC	48	48	0	39	12
Esophageal carcinoma	ESCA	173	162	11	140	109
Glioblastoma multiforme	GBM	173	168	5	102	547
Head and neck squamous cell carcinoma	HNSC	546	502	44	330	281
Kidney chromophobe	KICH	89	65	24	160	22
Kidney clear cell carcinoma	KIRC	607	535	72	638	341
Kidney papillary cell carcinoma	KIRP	321	289	32	313	65
Acute myeloid leukemia	LAML	151	151	0	168	458
Brain lower grade glioma	LGG	529	529	0	392	141
Liver hepatocellular carcinoma	LIHC	424	374	50	275	188
Lung adenocarcinoma	LUAD	585	526	59	469	269
Lung squamous cell carcinoma	LUSC	550	501	49	403	354
Mesothelioma	MESO	86	86	0	12	74
Ovarian serous cystadenocarcinoma	OV	379	379	0	296	435
Pancreatic adenocarcinoma	PAAD	182	178	4	92	130
Pheochromocytoma and paraganglioma	PCPG	186	183	3	181	8
Prostate adenocarcinoma	PRAD	551	499	52	607	16
Rectum adenocarcinoma	READ	177	167	10	144	34
Sarcoma	SARC	265	263	2	175	112
Skin cutaneous melanoma	SKCM	472	471	1	239	224
Stomach adenocarcinoma	STAD	407	375	32	292	210
Testicular germ cell tumor	TGCT	156	156	0	135	4
Thyroid carcinoma	THCA	568	510	58	592	22
Thymoma	THYM	121	119	2	126	12
Uterine corpus endometrioid carcinoma	UCEC	583	548	35	490	100
Uterine carcinosarcoma	UCS	56	56	0	21	40
Uveal melanoma	UVM	80	80	0	57	23

To facilitate the present intertumor and pan-cancer analyses, the gene expression levels were normalized to that of TBP (TATA box-binding protein). When investigating the differences in gene expression between tumor samples for different cancers and normal samples, we excluded types of cancer for which there were fewer than five associated normal-tissue samples. Applying these exclusion criteria resulted in 15 types of cancer being excluded; the remaining 18 types were BLCA, BRCA, CHOL, COAD, ESCA, GBM, HNSC, KICH, KIRC, KIRP, LIHC, LUAD, LUSC, PRAD, READ, STAD, THCA, and UCEC. When investigating the relationships between the gene expression levels of members of the TXNRD and GPX families and the overall survival of patients, we used tumor samples from all patients in the survival analysis because the survival information of patients was available for all 33 types of cancer.

### Analysis of the tumor microenvironment

The present tumor-microenvironment analysis was divided into three main correlation analyses: (1) between target genes and immune-cell subtypes, (2) between target genes and stromal- and immune-cell infiltration, and (3) between target genes and cancer stem cells (CSCs). There are six types of immune-cell infiltration in human tumors: C1 (wound healing), C2 (IFN-γ dominant), C3 (inflammatory), C4 (lymphocyte depleted), C5 (immunologically quiet), and C6 (TGF-β dominant) [64]. These different immune-cell subtypes are closely related to prognoses and may provide clues for the development of future immunotherapies [65]. We first analyzed the relationship between different immune-cell subtypes and overall patient survival. In order to understand the relationship between the gene members of the TXNRD and GPX families and immune-system components, we analyzed the relationship between the gene expression levels and immune-cell subtypes in TCGA pan-cancer data. The stromal- and immune-cell scores calculated using the ESTIMATE algorithm were used to assess the level of invasion of stromal and immune cells in different tumor types [66, 67]. This analysis was based on the interpretation of gene expression profiles retrieved from TCGA expression data (http://bioinformatics.mdanderson.org/estimate/). Cancer progression is often accompanied by the gradual loss of cell differentiation phenotypes and the acquisition of progenitor and stem-like characteristics, which in this study were measured based on RNAss and DNAss [20].

### Drug sensitivity analysis

We also investigated the correlation between transcript expression level of GPX and TXNRD genes and drug sensitivity. The NCI-60 is a panel of 60 human cancer cell lines used by the Developmental Therapeutics Program (DTP) of the U.S. National Cancer Institute to screen > 100,000 compounds plus natural products since 1990 [68, 69]. The NCI-60 database contains information on 60 different cancer cell lines related to 9 different tumor types. Nci-60 data have been widely used in cancer-related research and bioinformatics analysis, and have been widely recognized [70]. More detailed information about the NCI-60 data can be obtained from previous studies [71, 72]. CellMiner is a free tool that enables researchers to query NCI-60 data via the Web, including DNA,RNA, proteins and multiple molecular characterization at the pharmacological level [72] (https://discover.nci.nih.gov/cellminer/). In present study, the retrieved data of 60 cell lines including the GPX and TXNRD mRNA expression levels and z scores for cell sensitivity data (GI50) were downloaded from the CellMiner. The drug response of 262 FDA approved or drugs on clinical trials were used in the correlation analysis.

### Statistical analyses

The Wilcoxon rank-sum test was used to compare gene expression levels between the normal and tumor samples. The expression levels of different genes in different tumor types are presented using box plots. ANOVA was used to test the relationships between gene expression and immune-cell subtypes. The correlations of gene expression with the stromal-cell score, immune-cell score, stemness score, and drug sensitivity were measured using Spearman correlation. To reduce the likelihood of false positives in the correlation analysis, we used α = 0.05 as the examination standard [73].

In the survival analysis, univariate Cox proportional-hazards models were used to analyze the relationship between the expression of each gene and overall survival in cancer patients, with the results obtained presented as forest plots. All statistical tests were two-sided, and *p* values of < 0.05 were considered statistically significant. All statistical analyses were performed using R software (version 3.5.1).

## Supplementary Information

**Additional file 1: Table ****S1****.** Survival analysis of TXNRD and GPX gene expression with different cancer types

## Data Availability

The TCGA data set and the NCI60 cell line data were obtained from open databases. TCGA data set can be obtained from the following url: www.cancer.gov/about nci/organization/ccg/research/structural-genomics/tcga. NCI60 cell line data obtained from the link below: https://discover.nci.nih.gov/cellmine.

## Abbreviations

Se: Selenium
GPXs: Glutathione peroxidases
TXNRDs: Thioredoxin reductases
ANOVA: Analysis of variance
DNAss: Tumor stemness based on DNA methylation
r: Correlation coefficient
RNAss: Tumor cell stemness based on mRNA expression
Nrf2: Nuclear factor (erythroid-derived 2)-like 2
