# Supplementary material for: A pan-cancer study of selenoprotein genes as promising targets for cancer therapy
Source: BMC Med Genomics. 2021 Mar 11;14:78. doi: 10.1186/s12920-021-00930-1 (PMC7948377; doi:10.1186/s12920-021-00930-1)
Supplement: Supplementary file 1 — Additional file 1: Table S1. Survival analysis of TXNRD and GPX gene expression with different cancer types [file 12920_2021_930_MOESM1_ESM.docx]

Additional file 1: Table S1 Survival analysis of TXNRD and GPX gene expression with different cancer types

|  | GPX1 |  | GPX2 |  | GPX3 |  | GPX4 |  | TXNRD1 |  | TXNRD2 |  | TXNRD3 |  |
| --- | --- | --- | --- | --- | --- | --- | --- | --- | --- | --- | --- | --- | --- | --- |
|  | HR(95%CI) | *P* value | HR(95%CI) | *P* value | HR(95%CI) | *P* value | HR(95%CI) | *P* value | HR(95%CI) | *P* value | HR(95%CI) | *P* value | HR(95%CI) | *P* value |
| ACC | 1.157(0.685-1.952) | 0.586 | **133.711(1.06-16873.397)** | 0.047 | 0.844(0.637-1.118) | 0.237 | 0.795(0.474-1.33) | 0.382 | 0.771(0.503-1.183) | 0.234 | 1.01(0.519-1.966) | 0.977 | 1.341(0.512-3.514) | 0.551 |
| BLCA | 0.841(0.695-1.017) | 0.074 | 0.975(0.931-1.022) | 0.293 | 1.045(0.958-1.141) | 0.32 | 0.874(0.71-1.075) | 0.201 | **1.196(1.055-1.355)** | 0.005 | 0.95(0.777-1.162) | 0.62 | 1.092(0.803-1.486) | 0.574 |
| BRCA | **0.788(0.627-0.989)** | 0.04 | 0.982(0.845-1.141) | 0.81 | 1.034(0.918-1.166) | 0.579 | **0.777(0.624-0.967)** | 0.024 | **1.228(1.002-1.505)** | 0.048 | 0.955(0.702-1.299) | 0.769 | 1.082(0.789-1.482) | 0.625 |
| CESC | 0.777(0.594-1.018) | 0.067 | 0.968(0.889-1.053) | 0.446 | 1.037(0.901-1.194) | 0.616 | **0.691(0.513-0.93)** | 0.015 | 0.947(0.743-1.208) | 0.662 | 0.855(0.534-1.367) | 0.512 | 0.874(0.606-1.261) | 0.472 |
| CHOL | 0.679(0.278-1.657) | 0.395 | 1.106(0.822-1.49) | 0.506 | 0.986(0.693-1.403) | 0.937 | 0.977(0.502-1.903) | 0.946 | 1.241(0.791-1.947) | 0.348 | 1.126(0.321-3.945) | 0.853 | 0.936(0.332-2.635) | 0.9 |
| COAD | 1.132(0.871-1.472) | 0.353 | 0.961(0.814-1.135) | 0.64 | **1.234(1.033-1.476)** | 0.021 | 1.286(0.982-1.685) | 0.068 | 0.838(0.548-1.283) | 0.416 | 1.228(0.771-1.954) | 0.387 | 1.526(0.902-2.583) | 0.115 |
| DLBC | 0.584(0.244-1.397) | 0.226 | 3.932(0.362-42.689) | 0.261 | 0.862(0.524-1.419) | 0.559 | 0.538(0.215-1.342) | 0.183 | 0.768(0.324-1.819) | 0.548 | 0.589(0.164-2.122) | 0.418 | 0.037(0.001-2.384) | 0.121 |
| ESCA | 0.772(0.507-1.175) | 0.227 | 1.002(0.889-1.13) | 0.973 | 1.079(0.916-1.27) | 0.364 | 0.81(0.551-1.191) | 0.284 | 1.218(0.971-1.528) | 0.088 | 0.883(0.42-1.855) | 0.742 | 0.84(0.533-1.323) | 0.452 |
| GBM | 1.046(0.858-1.277) | 0.654 | 7.308(0.854-62.581) | 0.069 | 1.041(0.926-1.169) | 0.501 | 1.013(0.737-1.392) | 0.938 | 1.107(0.754-1.624) | 0.604 | 0.598(0.348-1.029) | 0.064 | 1.141(0.655-1.987) | 0.642 |
| HNSC | 0.901(0.745-1.09) | 0.282 | 1.053(0.998-1.111) | 0.06 | 0.989(0.898-1.09) | 0.827 | 0.893(0.747-1.069) | 0.217 | **1.146(1.038-1.265)** | 0.007 | 1.185(0.884-1.588) | 0.255 | 1.009(0.8-1.271) | 0.943 |
| KICH | 2.982(0.727-12.238) | 0.129 | **0.119(0.017-0.831)** | 0.032 | 0.714(0.421-1.209) | 0.21 | 0.458(0.08-2.617) | 0.38 | 2.71(0.838-8.77) | 0.096 | 1.025(0.163-6.451) | 0.979 | **15.104(1.243-183.56)** | 0.033 |
| KIRC | **1.371(1.135-1.655)** | 0.001 | 1.156(0.967-1.383) | 0.112 | **0.855(0.756-0.968)** | 0.013 | 1.077(0.869-1.334) | 0.498 | 1.241(0.963-1.599) | 0.096 | 0.861(0.58-1.277) | 0.457 | 0.815(0.511-1.299) | 0.389 |
| KIRP | **0.623(0.432-0.898)** | 0.011 | **1.178(1.042-1.332)** | 0.009 | 0.914(0.765-1.093) | 0.325 | 0.837(0.503-1.392) | 0.493 | **1.429(1.2-1.701)** | <0.001 | **0.148(0.071-0.31)** | <0.001 | 0.563(0.232-1.362) | 0.202 |
| LAML | **1.661(1.249-2.208)** | <0.001 | 0.766(0.507-1.156) | 0.204 | 0.722(0.459-1.137) | 0.16 | **2.111(1.41-3.161)** | <0.001 | 0.592(0.327-1.072) | 0.084 | 1.812(0.875-3.754) | 0.11 | 1.058(0.787-1.423) | 0.707 |
| LGG | **1.571(1.239-1.991)** | <0.001 | 0.816(0.206-3.224) | 0.772 | **0.87(0.787-0.961)** | 0.006 | 0.994(0.663-1.49) | 0.978 | **1.819(1.133-2.92)** | 0.013 | **0.454(0.272-0.758)** | 0.003 | 0.674(0.42-1.081) | 0.102 |
| LIHC | 1.116(0.901-1.383) | 0.316 | 1.039(0.967-1.117) | 0.298 | 1.023(0.906-1.157) | 0.71 | 1.266(0.971-1.651) | 0.081 | **1.369(1.177-1.593)** | <0.001 | 0.986(0.762-1.276) | 0.917 | 1.109(0.673-1.83) | 0.684 |
| LUAD | 1.041(0.876-1.238) | 0.648 | 1.048(0.999-1.099) | 0.055 | **0.839(0.737-0.956)** | 0.008 | 0.94(0.777-1.136) | 0.52 | **1.135(1.032-1.249)** | 0.009 | 0.91(0.702-1.181) | 0.479 | 0.891(0.609-1.304) | 0.553 |
| LUSC | 1.027(0.838-1.259) | 0.795 | 0.983(0.937-1.031) | 0.473 | **1.172(1.055-1.302)** | 0.003 | 1.123(0.889-1.419) | 0.329 | 1.013(0.928-1.106) | 0.775 | 0.98(0.729-1.316) | 0.892 | 1.065(0.865-1.311) | 0.555 |
| MESO | 0.86(0.599-1.236) | 0.415 | 1.38(0.252-7.54) | 0.71 | 0.946(0.765-1.169) | 0.606 | 1.169(0.813-1.681) | 0.401 | 0.975(0.717-1.324) | 0.869 | 1.213(0.783-1.88) | 0.387 | 1.202(0.597-2.418) | 0.606 |
| OV | 0.924(0.755-1.131) | 0.441 | 0.77(0.529-1.12) | 0.172 | 1.064(0.988-1.146) | 0.102 | 0.959(0.793-1.161) | 0.67 | 1.093(0.858-1.393) | 0.473 | 0.957(0.704-1.3) | 0.778 | 1.022(0.792-1.321) | 0.865 |
| PAAD | 1.206(0.878-1.656) | 0.247 | 1.073(0.951-1.21) | 0.252 | **0.771(0.663-0.897)** | 0.001 | 0.73(0.523-1.018) | 0.064 | 1.446(0.976-2.142) | 0.066 | **0.472(0.251-0.886)** | 0.019 | **2.734(1.409-5.304)** | 0.003 |
| PCPG | 0.805(0.275-2.359) | 0.693 | 1.3(0.188-9.001) | 0.79 | 0.728(0.483-1.099) | 0.131 | 0.887(0.192-4.102) | 0.878 | 0.564(0.183-1.734) | 0.317 | 0.241(0.034-1.712) | 0.155 | 1.491(0.23-9.684) | 0.676 |
| PRAD | 0.676(0.271-1.682) | 0.399 | 1.087(0.613-1.93) | 0.775 | 0.69(0.418-1.14) | 0.148 | 0.504(0.214-1.189) | 0.118 | 1.636(0.68-3.936) | 0.272 | **0.462(0.254-0.841)** | 0.012 | 1.147(0.191-6.897) | 0.881 |
| READ | 1.074(0.555-2.078) | 0.833 | 1.663(0.934-2.962) | 0.084 | **1.325(1.012-1.736)** | 0.041 | 1.112(0.616-2.007) | 0.725 | 0.679(0.306-1.506) | 0.341 | 0.723(0.278-1.877) | 0.505 | 0.439(0.165-1.165) | 0.098 |
| SARC | 0.907(0.728-1.131) | 0.387 | 0.935(0.542-1.613) | 0.809 | 0.941(0.857-1.033) | 0.201 | 0.954(0.749-1.215) | 0.701 | 0.895(0.71-1.129) | 0.349 | 0.941(0.676-1.309) | 0.717 | 1.464(0.963-2.226) | 0.074 |
| SKCM | 0.915(0.787-1.063) | 0.245 | **1.382(1.184-1.612)** | <0.001 | 0.984(0.908-1.065) | 0.682 | 1.029(0.82-1.291) | 0.806 | 1.02(0.839-1.242) | 0.839 | **1.528(1.195-1.955)** | 0.001 | 1.029(0.785-1.348) | 0.838 |
| STAD | 1.17(0.937-1.46) | 0.165 | 0.926(0.843-1.016) | 0.105 | **1.245(1.101-1.409)** | <0.001 | 1.155(0.913-1.46) | 0.23 | 0.968(0.738-1.269) | 0.814 | 0.915(0.592-1.415) | 0.69 | 1.063(0.764-1.48) | 0.715 |
| TGCT | 2.768(0.489-15.685) | 0.25 | 0.923(0.537-1.588) | 0.773 | 0.628(0.17-2.329) | 0.487 | 1.163(0.188-7.186) | 0.871 | 1.549(0.361-6.641) | 0.556 | 0.676(0.068-6.758) | 0.739 | 0.354(0.031-4.083) | 0.405 |
| THCA | **0.389(0.161-0.94)** | 0.036 | 1.109(0.371-3.311) | 0.854 | 1.045(0.648-1.685) | 0.857 | **0.348(0.138-0.876)** | 0.025 | **1.95(1.202-3.162)** | 0.007 | 1.142(0.282-4.625) | 0.853 | **8.097(1.229-53.33)** | 0.03 |
| THYM | 2.055(0.658-6.424) | 0.215 | 1.102(0.788-1.541) | 0.571 | 1.021(0.675-1.545) | 0.921 | 1.276(0.456-3.572) | 0.643 | 0.727(0.282-1.87) | 0.508 | 1.164(0.295-4.594) | 0.828 | **4.811(1.275-18.15)** | 0.02 |
| UCEC | **0.767(0.608-0.968)** | 0.026 | 0.95(0.856-1.055) | 0.338 | 1.08(0.971-1.2) | 0.157 | **0.669(0.527-0.849)** | 0.001 | 1.117(0.906-1.378) | 0.3 | 0.765(0.502-1.165) | 0.211 | **1.625(1.049-2.519)** | 0.03 |
| UCS | 1.172(0.726-1.89) | 0.516 | 1.225(0.788-1.905) | 0.368 | 1.048(0.768-1.429) | 0.768 | 1.337(0.798-2.238) | 0.27 | 1.161(0.592-2.277) | 0.664 | 0.876(0.463-1.657) | 0.683 | 0.921(0.478-1.775) | 0.806 |
| UVM | **3.241(1.303-8.062)** | 0.011 | **6.08(1.126-32.843)** | 0.036 | **0.732(0.542-0.988)** | 0.041 | 2.106(0.915-4.844) | 0.08 | 1.721(0.865-3.426) | 0.122 | **4.582(1.864-11.263)** | 0.001 | 0.469(0.179-1.225) | 0.122 |
